# Effect of Silica Sol on the Preparation and Oxidation Resistance of MoSi_2_@SiO_2_

**DOI:** 10.3390/ma18133203

**Published:** 2025-07-07

**Authors:** Linlin Guo, Jinjun Zhang, Chengpeng Miao, Shuang Feng, Xiaozhen Fan, Haiyan Du, Jiachen Liu, Mingchao Wang

**Affiliations:** 1 College of Chemistry and Chemical Engineering, Cangzhou Normal University, Cangzhou 061001, China; 2 Key Lab of Advanced Ceramics and Machining Technology of the Ministry of Education, School of Materials Science and Engineering, Tianjin University, Tianjin 300350, China; 3 College of Science, Civil Aviation University of China, Tianjin 300300, China

**Keywords:** MoSi_2_, silica sol, core-shell structure, oxidation resistance

## Abstract

The limited oxidation resistance of MoSi_2_ between 400 °C and 600 °C restricts its aerospace applications. This study develops a silica-sol derived core-shell MoSi_2_@SiO_2_ composite to enhance the low-temperature oxidation resistance of MoSi_2_. Acidic, neutral, and basic silica sols were systematically applied to coat MoSi_2_ powders through sol-adsorption encapsulation. Two pathways were used, one was ethanol-mediated dispersion, and the other was direct dispersion of MoSi_2_ particles in silica sol. Analysis demonstrated that ethanol-mediated dispersion significantly influenced the coating efficiency and oxidation resistance, exhibited significantly decreased coating weight gains (maximum 27%) and increased oxidation weight gains (10–20%) between 340 °C and 600 °C compared with direct dispersion of MoSi_2_ particles with silica sol, ascribe to the kinetic inhibition of hydroxyl group condensation and steric hindrance of MoSi_2_-silica sol interface interactions of ethanol. Systematic investigation of silica sol encapsulation of MoSi_2_ revealed critical correlations between colloid properties and oxidation resistance of MoSi_2_@SiO_2_. Basic silica sol coated MoSi_2_ (BS-MoSi_2_) exhibits the lowest coating efficiency (coating weight gain of 7.74 ± 0.06%) as well as lowest oxidation weight gain (18.45%) between 340 °C and 600 °C compared with those of acid and neutral silica sol coated MoSi_2_ (AS-MoSi_2_ and NS-MoSi_2_), arises from optimal gelation kinetics, enhanced surface coverage via reduced agglomeration, and suppressed premature nucleation through controlled charge interactions under alkaline conditions.

## 1. Introduction

MoSi_2_ has emerged as a promising high-temperature structural material with multifunctional applications in ceramic, metallic, and C/C composite-based coatings. Its exceptional infrared emissivity makes it indispensable as the primary emittance agent in radiative thermal protection coatings [1,2,3]. The material’s inherent oxidation resistance at high temperatures (>800 °C) positions MoSi_2_ as the principal constituent of oxidation-resistant coatings for metals and C/C composites [4,5]. Furthermore, MoSi_2_, serving as self-healing particles, can oxidize and form a glass-like silicon dioxide (SiO_2_) layer, which can fill the cracks in thermal barrier coatings (TBCs) [6,7]. However, critical limitations persist: conventional MoSi_2_-containing coatings need to be applied in high-temperature conditions, and the material exhibits pulverization oxidation characteristics due to volume expansion within 400–600 °C [8,9]. Current research efforts focus on developing composite modification strategies to simultaneously suppress oxidation-induced decohesion and maintain mechanical integrity under service conditions. Addressing these critical limitations is essential for advancing MoSi_2_-based thermal protection coatings in aerospace.

Current strategies for enhancing MoSi_2_ oxidation resistance primarily focus on two material engineering paradigms: (1) alloying design through the addition of refractory metals (e.g., Al, Nb) forming intermetallic compounds [10,11], and (2) composite reinforcement using secondary phases (e.g., SiC, ZrB_2_) [12,13]. While these approaches effectively improve oxidation resistance in bulk composites, their application in powder-based materials with porous and loose structures faces critical limitations. For MoSi_2_ materials, their high-temperature oxidation resistance comes from the formation of a dense SiO_2_ protective layer at high temperatures, which prevents oxygen from entering and further oxidation of the internal materials [14]. Therefore, generating a protective coating through pre-oxidation has become an important way of improving the oxidation resistance of MoSi_2_ materials [15,16]. However, for MoSi_2_ powder, strict conditions are required to generate an in situ protective coating through the pre-oxidation method [17]. Advances in surface modification engineering propose an alternative pathway: chemical synthesis of core-shell structured MoSi_2_@SiO_2_ particles [18]. This bottom-up approach enables independent control over shell thickness and crystallinity through sol-gel parameters (pH, precursor ratio) while avoiding thermal degradation of the core material [19].

The use of SiO_2_ as a protective shell for MoSi_2_ material mainly lies in its high stability and optical transparency, which can protect MoSi_2_ from oxidation without affecting its emissivity. Xiang’s research suggests that the emissivity of both layered structures with SiO_2_ covered on the surface of MoSi_2_ and composites with SiO_2_ mixed with MoSi_2_ is enhanced significantly and demonstrates that oxides play a dual role in determining the emissivity of MoSi_2_, i.e., anti-reflective agents and binders [1]. Perhydropolysilazane, tetraethyl orthosilicate (TEOS) and silica sol can be used as precursors for generating silica shells. In patent US6391383, the SiB_4_ is dispersed in perhydropolysilazane, and after heat treatment at 400 °C, a SiO_2_ film is formed on the surface of SiB_4_ to prevent oxidation and serve as a reaction barrier, effectively improving the emissivity of the coating [20]. Perhydropolysilazane can be converted to SiO_2_ at lower temperatures, but it is expensive. Chen et al. prepared MoSi_2_@SiO_2_ particles via the tetraethyl orthosilicate (TEOS) hydrolysis self-assembly method with tetrabutyl ammonium bromide (TBAB) as an electrostatic adsorbent after heat treatment at 1200 °C [21]. Strict control conditions are required for the hydrolysis and polymerization of TEOS. In comparison, silica sol is an ideal precursor for forming silica shells because of its low price and easy control of the gel process [22,23]. Silica sol is a highly dispersed colloid of poly silicic acid with water as the dispersing medium, which is a heterogeneous inorganic polymer with good adhesion and film-forming properties [24]. In our previous work, core-shell structured MoSi_2_@SiO_2_ was prepared with the sol-gel process followed by pre-oxidation at 800 °C, in which a dense coating layer formed as the shell. It showed less weight gains (<1 wt%) than those prepared with the direct preoxidation method (<1.6 wt%) after cyclic-isothermal oxidation at 400–600 °C for 12 h [17,19], indicating that the sol-adsorption coating process played an important role in improving the oxidation resistance of MoSi_2_@SiO_2_, and, therefore, the factors (such as dispersant and pH) affecting the properties of silica sol, as well as the gel coating MoSi_2_@SiO_2_, need to be carefully studied. In this paper, the influence of the sol-adsorption process (such as dispersant and pH) on shell formation, as well as the oxidation resistance of core-shell MoSi_2_@SiO_2_, was mainly focused on without high-temperature pre-oxidation treatment. To reveal the influence of the silica gel coating on the oxidation resistance of MoSi_2_@SiO_2_, three kinds of silica sol were used as the precursor to prepare MoSi_2_@SiO_2_ particles, and the effect of silica sols on the oxidation resistance of MoSi_2_@SiO_2_ was deeply investigated.

## 2. Materials and Methods

MoSi_2_ powder (Eno Material, Qinhuangdao, China) with a median particle size (D50) of 3.721 μm was used as raw material. Aqueous silica sol was commercially available (Jinghuo Technique Glass Co., Ltd., Dezhou, China) with detailed parameters in Table 1. The MoSi_2_@SiO_2_ composites were fabricated through a sol-adsorption process using acidic, neutral, and basic silica sols (pH values detailed in Table 1) as precursors. Two pathways were used: one was ethanol-mediated dispersion, and the other was direct dispersion of MoSi_2_ particles in silica sol. Precisely weighed MoSi_2_ powders (±0.0001 g accuracy) received a certain amount of respective silica sols with a mass ratio of 1:3 (MoSi_2_:SiO_2_) via pipetting, followed by ultrasonic dispersion (40 kHz, 25 °C) for 10 min to achieve homogeneous distribution. In the ethanol-mediated dispersion process, the amount of ethanol used is 2.5 mL/g, depending on the mass of MoSi_2_. The mixture underwent magnetic stirring (800 rpm, 25 ± 1 °C) for 4 h to form stable suspensions. Subsequent phase separation involved centrifugation (3000 rpm, 20 °C, 30 min) and 24 h sedimentation in sealed containers. The supernatant was decanted, and the residue was subjected to sequential drying: 30 min at 65 °C for solvent removal followed by 4 h at 105 °C for complete dehydration. The resultant xerogels were designated as AS-MoSi_2_ (acidic), NS-MoSi_2_ (neutral), and BS-MoSi_2_ (basic) based on colloid pH characteristics.

The coating efficiency was quantified through gravimetric analysis using Equation (1):

(1) $$w/\%=\frac{m_{2}-m_{1}}{m_{1}}\times 100\%,$$

where *w* represents the coating weight gain rate (%), *m*_1_ indicates the pre-treatment MoSi_2_ mass (g), and *m*_2_ denotes the post-treatment composite mass (g). All mass measurements were conducted using the analytical balance (±0.1 mg precision).

The surface chemical groups of AS-MoSi_2_, NS-MoSi_2_, and BS-MoSi_2_ were characterized by Fourier transform infrared spectroscopy (FTIR, Nicolet iS5, Thermo Scientific, Waltham, MA, USA) employing the KBr pellet technique in transmission mode. Phase evolution during oxidation was analyzed through X-ray Diffraction (XRD, TD-3500 Tongda, Dandong, China) with Cu Kα radiation (λ = 1.5406 Å). Microstructural features and elemental distribution were examined using scanning electron microscopy (SEM, TM3030, Hitachi, Tokyo, Japan) coupled with energy-dispersive spectroscopy (EDS). The specific surface area and pore structure of the samples were determined using a specific surface area analyzer (Kobu X1000, Builder, Hangzou, China).

Thermogravimetric analysis (TG, HCT-4, Hengjiu, Beijing, China) was conducted to evaluate non-isothermal oxidation kinetics under an air atmosphere (heating rate 10 °C/min, 40–1100 °C). For isothermal cyclic oxidation evaluation, disc specimens (diameter 1.33 cm, thickness 0.25 ± 0.001 cm) were prepared by cold pressing 0.5000 ± 0.0010 g powder samples at 20 kPa for 10 s. The cyclic oxidation tests at 400 °C, 500 °C, 600 °C, and 700 °C followed a standardized procedure: samples were loaded into preheated furnaces, held at the target temperature for 1 h, then quenched to room temperature. Mass variation was quantified according to Equation (2) using an analytical balance (±0.1 mg precision) after each oxidation cycle.

(2) $$w_{o}/\%=\frac{m_{h}-m_{0}}{m_{0}}\times 100\%$$

where *w_o_* is the oxidation weight change percent (%), *m_h_* is the weight of samples after isothermal cyclic oxidation for a certain time (g), and *m*_0_ is the initial sample weight (g).

## 3. Results

### 3.1. Effects of Ethanol-Mediated Process on Coating Efficiency and Oxidation Resistance

**Figure 1:**
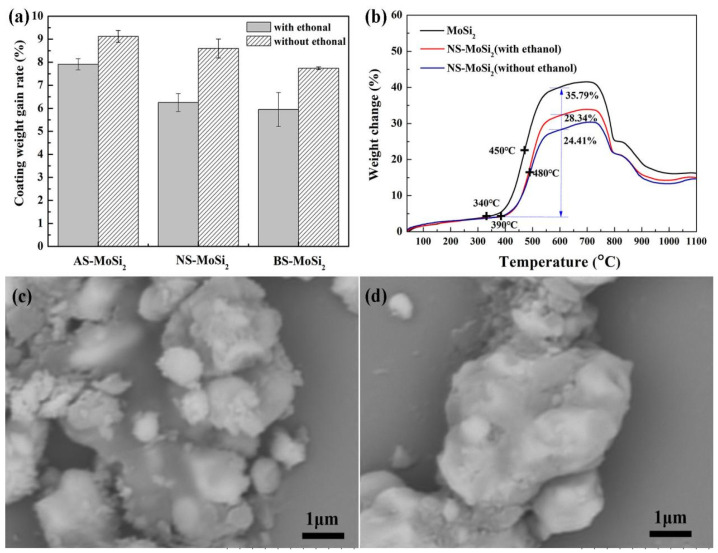
(**a**) Coating efficiency of AS-MoSi_2_, NS-MoSi_2_, and BS-MoSi_2_ fabricated via ethanol-mediated dispersion vs. direct mixing. (**b**) Comparative thermogravimetric (TG) curves of MoSi_2_ and NS-MoSi_2_ (ethanol-mediated vs. direct mixing). (**c**,**d**) Scanning electron microscopy (SEM) microstructures of NS-MoSi_2_ under different processing routes: (**c**) ethanol-mediated and (**d**) direct mixing.

In order to analyze the effect of ethanol-mediated dispersant, a comparison was made between the xerogel complexes obtained from the two pathways. Samples obtained from ethanol-mediated dispersion are labeled as “with ethanol”, and direct mixing of MoSi_2_ with silica sol is labeled as “without ethanol”. As shown in Figure 1a, all AS-MoSi_2_, NS-MoSi_2_, and BS-MoSi_2_ samples fabricated via ethanol-mediated dispersion exhibited significantly reduced coating efficiency (CE) values when compared with direct mixing. Notably, this solvent-dependent CE discrepancy reached 27% maximum difference for NS-MoSi_2_ systems, suggesting that ethanol-mediated dispersion plays an important role in oxide layer formation.

When MoSi_2_ is directly mixed with silica sol, interfacial water molecules react with MoSi_2_ surfaces to form oxygen-containing functional groups (hydroxyl –OH and Si–O bonds). Surface hydroxyl species (Si–OH) undergo condensation polymerization through dehydration reaction (Reaction (1)):

Si–OH (MoSi_2_) + Si–OH → Si–O–Si (MoSi_2_) + H_2_O (R1)

During solvent evaporation, these Si–OH groups interconnect to construct a three-dimensional SiO_2_ network on MoSi_2_ surfaces.

In ethanol-mediated systems, particle interactions follow DLVO theory [25,26]. Ethanol’s weak chemical adsorption via ethoxy (–OC_2_H_5_) and trace hydroxyl groups exhibits lower reactivity compared with aqueous systems [25], resulting in suppressed premature Si–O–Si network formation. Ethanol’s dual role includes: kinetic inhibition of hydroxyl group condensation and steric hindrance of MoSi_2_-silica sol interface interactions. These effects of ethanol explain the reduced coating efficiency (CE) observed in samples fabricated via ethanol-mediated dispersion system, where slower reaction kinetics lead to 10–30% lower coating weight gain rate values versus direct mixing processing.

Thermogravimetric (TG) analysis revealed distinct oxidation resistance trends among AS-MoSi_2_, NS-MoSi_2_, and BS-MoSi_2_ prepared via ethanol-mediated dispersion versus direct mixing. All xerogel composites exhibited a similar trend but reduced mass gain compared to pristine MoSi_2_, with ethanol-free preparations showing 10–20% lower weight gains between 340 °C and 600 °C than their ethanol-dispersed counterparts. As shown in Figure 1b, the NS-MoSi_2_ system demonstrated delayed oxidation kinetics: oxidation initiation shifted from 340 °C (MoSi_2_) to 390 °C, with peak reaction rates occurring at 480 °C (vs. 450 °C for MoSi_2_).

MoSi_2_ undergoes an oxidation reaction as shown in Reaction (2), with the theoretical weight gain of the complete reaction being 73.63%.

2MoSi_2_(s) + 7O_2_(g) = 4SiO_2_(s) + 2MoO_3_(s) (R2)

As illustrated in Figure 1b, the TG curves reveal that there exists an upward trend of the weight change between 340 °C and 750 °C, but when the temperature exceeds the volatilization temperature of the oxidation product MoO_3_ (750 °C), the weight change begins to decline. Notably, the weight gains of MoSi_2_, NS-MoSi_2_ (with ethanol), and NS-MoSi_2_ (without ethanol) between 340 °C and 600 °C are 35.79%, 28.34%, and 24.44%, respectively. These data indicate that NS-MoSi_2_ experiences a postponed onset of oxidation and reduced oxidation weight gain compared with unmodified MoSi_2_, confirming that the gel coating enhances oxidation resistance of the coated composites to some degree. Furthermore, the NS-MoSi_2_ sample prepared through the direct mixing of MoSi_2_ with silica sol exhibits superior oxidation resistance compared with the sample where MoSi_2_ was first dispersed in ethanol prior to silica sol addition. This difference is likely attributable to the microstructural characteristics visible in Figure 1c,d.

While ethanol dispersion theoretically should reduce particle agglomeration and create more uniform particle exposure for silica sol polymerization, the slower polymerization kinetics under ethanol conditions result in a less dense protective film formation, as evidenced by the SEM images (Figure 1c). The direct mixing method appears to produce a more continuous and uniform coating layer (Figure 1d), leading to improved oxidation protection.

These findings underscore the significant influence of preparation methodology on protective coating characteristics and resulting oxidation resistance. For optimal antioxidant performance in practical applications, the direct mixing method of MoSi_2_ and silica sol is recommended for preparing MoSi_2_@SiO_2_ composites.

### 3.2. Effect of Silica Sol on the Oxidation Resistance of Gel Coating MoSi_2_

#### 3.2.1. Effect of Silica Sol on Coating Formation

As illustrated in Figure 1a, there is a clear correlation between the coating weight gain rate and the type of silica sol used. The AS-MoSi_2_ sample exhibits the highest coating weight gain rate of 9.12 ± 0.26%, followed by NS-MoSi_2_ of 8.60 ± 0.41%, while BS-MoSi_2_ demonstrates the lowest value of 7.74 ± 0.06%.

The zeta potential of MoSi_2_ in aqueous solution reveals that the surface charge of MoSi_2_ progressively becomes more negative as the pH increases from 3 to 11 [27]. This negative surface charge characteristic plays a critical role in the coating formation process when MoSi_2_ is dispersed in silica sol. The surface charge generation on MoSi_2_ can be attributed to the surface reaction described by Reaction (3):

SiOH (MoSi_2_) + H_2_O = SiO^−^ (MoSi_2_) + H_3_O^+^ (R3)

The isoelectric point of pure silica is typically around pH 3 [25]. Consequently, in acidic silica sol environments (pH < 3), the silicon hydroxyl groups undergo protonation according to Reaction (4), resulting in positively charged silica particles (Si–OH_2_^+^).

SiOH + H^+^ = SiOH_2_^+^ (R4)

In neutral and alkaline silica sol conditions (pH > 3), deprotonation occurs as shown in Reactions (5) and (6). These reactions lead to negatively charged silica surfaces (Si–O^−^).

SiOH + H_2_O = SiO^−^ + H_3_O^+^ (R5)

SiOH + OH^−^ = SiO^−^ + H_2_O (R6)

During the sol-gel process, condensation reactions represent the critical step for coating formation on MoSi_2_ particles. The mechanisms of condensation are based on two steps: protonation/deprotonating (according to the pH), and then reaction of the protonated/deprotonated particle with the raw species [28]. These condensation mechanisms depend on the surface charge characteristics, which are pH-dependent:

In acidic conditions, the positively charged silica sol particles (Si–OH_2_^+^) are strongly attracted to the negatively charged MoSi_2_ surface (Si–O^−^) through electrostatic interactions. This results in efficient adsorption and deposition of silica onto MoSi_2_ particles, yielding the highest coating weight gain rate observed. In neutral conditions, the silica sol particles exhibit reduced surface charge, leading to poorer colloidal stability and increased tendency for particle aggregation. This results in decreased coating efficiency and lower weight gain compared to acidic conditions. While in alkaline conditions, the silica sol particles carry negative surface charges (Si–O^−^), which create electrostatic repulsion with the similarly charged MoSi_2_ surface (Si–O^−^). This significantly reduces adsorption efficiency and produces the lowest coating weight gain rate.

#### 3.2.2. Surface Characteristics and Thermogravimetric Analysis

**Figure 2:**
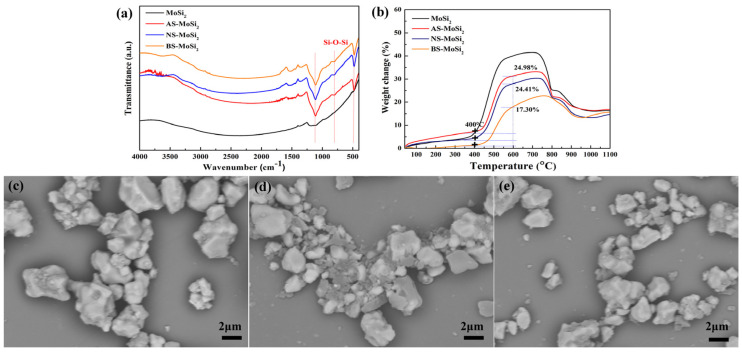
FTIR spectrum with red lines indicating the vibration frequency locations of Si–O–Si (**a**) and TG (**b**) curves of MoSi_2_, AS-MoSi_2_, NS-MoSi_2_, and BS-MoSi_2_, and SEM images of AS-MoSi_2_ (**c**), NS-MoSi_2_ (**d**) and BS-MoSi_2_ (**e**).

To investigate the surface chemical characteristics of MoSi_2_ before and after silica coating treatments, Fourier transform infrared spectroscopy (FTIR) was employed, and the spectra are shown in Figure 2a. The unmodified MoSi_2_ sample exhibited negligible absorption features across the measured spectral range (4000–400 cm^−1^), indicating minimal surface functionalization. In contrast, the coated samples (AS-MoSi_2_, NS-MoSi_2_, and BS-MoSi_2_) all displayed distinct absorption bands at approximately 1100 cm^−1^, 800 cm^−1^, and 475 cm^−1^, which correspond to the asymmetric stretching, symmetric stretching, and bending vibrations of the Si–O–Si network structure [29,30]. These findings confirm the successful formation of silica-based coatings on the MoSi_2_ particle surfaces.

The thermogravimetric analysis (Figure 2b) demonstrates that silica coating substantially enhances the oxidation resistance of MoSi_2_. The oxidation onset temperature increased from approximately 340 °C for pristine MoSi_2_ to about 400 °C for all coated derivatives. Between 340 °C and 600 °C, the weight gains due to oxidation were quantified as 31.58%, 28.34%, and 18.45% for AS-MoSi_2_, NS-MoSi_2_, and BS-MoSi_2_, respectively. This trend (BS-MoSi_2_ < NS-MoSi_2_ < AS-MoSi_2_) indicates that BS-MoSi_2_ exhibits superior antioxidant capacity, likely due to differences in coating microstructure and density.

Scanning electron microscopy (SEM) images (Figure 2c–e) reveal pronounced morphological differences among the silica-coated MoSi_2_ variants, which correlate with their respective oxidation behaviors. The AS-MoSi_2_ sample shows relatively large aggregated particles with uneven surface coverage and some interparticle connectivity (Figure 2c). The NS-MoSi_2_ displays partial surface coating with observable silica aggregate formations (Figure 2d). In contrast, BS-MoSi_2_ exhibits the most uniform morphology (Figure 2e), with well-dispersed particles and a consistent gel-like coating layer that appears to fully encapsulate the MoSi_2_ core. These structural variations provide clear visual evidence explaining the differences in oxidation resistance observed in the thermal analysis.

#### 3.2.3. Oxidation Behavior of the Silica-Coated MoSi_2_

**Figure 3:**
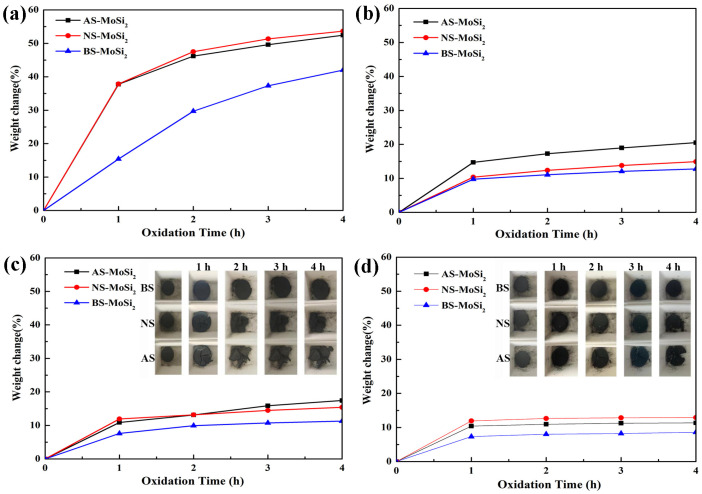
The isothermal cyclic oxidation behavior of AS-MoSi_2_, NS-MoSi_2_, and BS-MoSi_2_ at 400 °C (**a**), 500 °C (**b**), 600 °C (**c**), and 700 °C (**d**), with physical images of the samples in (**c**,**d**).

**Figure 4:**
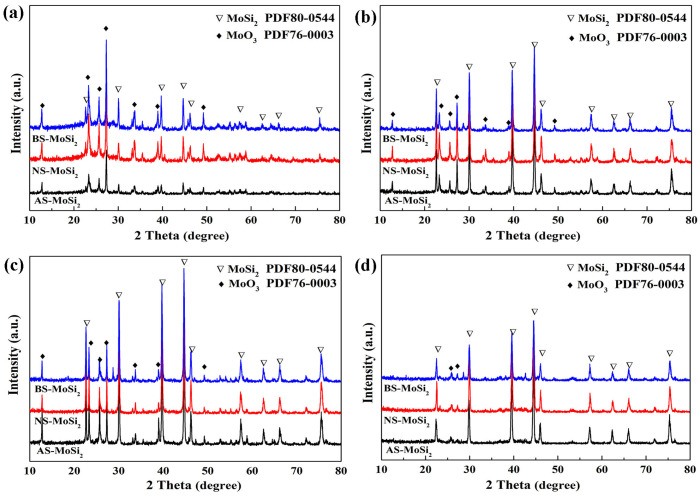
XRD patterns of AS-MoSi_2_, NS-MoSi_2_, and BS-MoSi_2_ after isothermal cyclic oxidation at 400 °C (**a**), 500 °C (**b**), 600 °C (**c**), and 700 °C (**d**) for 4 h.

The isothermal cyclic oxidation behavior of AS-MoSi_2_, NS-MoSi_2_, and BS-MoSi_2_ was systematically investigated at temperatures ranging from 400 °C to 700 °C. As illustrated in Figure 3, the weight change kinetics demonstrated distinct temperature-dependent patterns for all three coating types. XRD patterns of the AS-MoSi_2_, NS-MoSi_2_, and BS-MoSi_2_ after isothermal oxidation tests for 4 h are shown in Figure 4.

At 400 °C (Figure 3a), AS-MoSi_2_ and NS-MoSi_2_ exhibited relatively rapid high oxidation weight gains compared to that of BS-MoSi_2_, with weight gains exceeding 40% after 4 h of exposure. This significant weight increase was accompanied by the visible pulverization and volumetric expansion of the samples, indicating vigorous oxidation reactions at this relatively low temperature. Diffraction peaks corresponding to MoO_3_ (PDF#76-0003) derived from the oxidation of MoSi_2_ can be detected in all three samples tests at 400 °C, and the diffraction peaks corresponding to MoSi_2_ (PDF#80-0544) are relatively weak, especially for AS-MoSi_2_ (Figure 4a), resulting from the oxidation of MoSi_2_ at this temperature.

As the temperature increased to 500 °C (Figure 3b), the oxidation rate decreased noticeably for all samples compared to those at 400 °C, with weight gains lower than 20%. Notably, BS-MoSi_2_ consistently showed the lowest weight gain across all time points, while AS-MoSi_2_ displayed the most pronounced oxidative degradation, particularly in the later stages of exposure. Although the peak reaction rates occur near 500 °C according to the TG analysis (Figure 2b), diffraction peaks corresponding to MoSi_2_ after isothermal cyclic oxidation at 500 °C are stronger than those at 400 °C (Figure 4b). The samples were oxidized less intensely at 500 °C than those at 400 °C, which may be due to the less volume expansion at the initial stage of oxidation, avoiding more channels for oxygen to enter.

At higher temperatures of 600 °C and 700 °C, the oxidation kinetics exhibited a two-stage behavior: an initial rapid weight gain phase within the first hour, followed by a gradual stabilization period (Figure 3c,d). According to thermal analysis (Figure 2b), the TG curves at 600–700 °C are close to the plateau, indicating a slow oxidation reaction rate at this stage. Among the three samples, BS-MoSi_2_ maintained the lowest weight gain throughout the entire exposure period (<10%), with only minor surface cracking observed in its visual micrographs (Figure 3c,d). In contrast, AS-MoSi_2_ and NS-MoSi_2_ samples displayed increasingly severe surface cracking and structural degradation upon oxidation time, particularly in AS-MoSi_2_. In XRD patterns, weak diffraction peaks corresponding to MoO_3_ (PDF#76-0003) were detected in sample tests at 700 °C compared with those at 600 °C (Figure 4c,d), which may be attributed to the volatilization of MoO_3_ at this temperature.

The observed differences in oxidation behavior among AS-MoSi_2_, NS-MoSi_2_, and BS-MoSi_2_ can be attributed to variations in their surface adsorption and the consequent effects on protective oxide layer formation. The enhanced oxidation resistance of BS-MoSi_2_ is likely related to its unique compositional characteristics that promote the formation of more adherent and continuous protective oxide scales.

**Figure 5:**
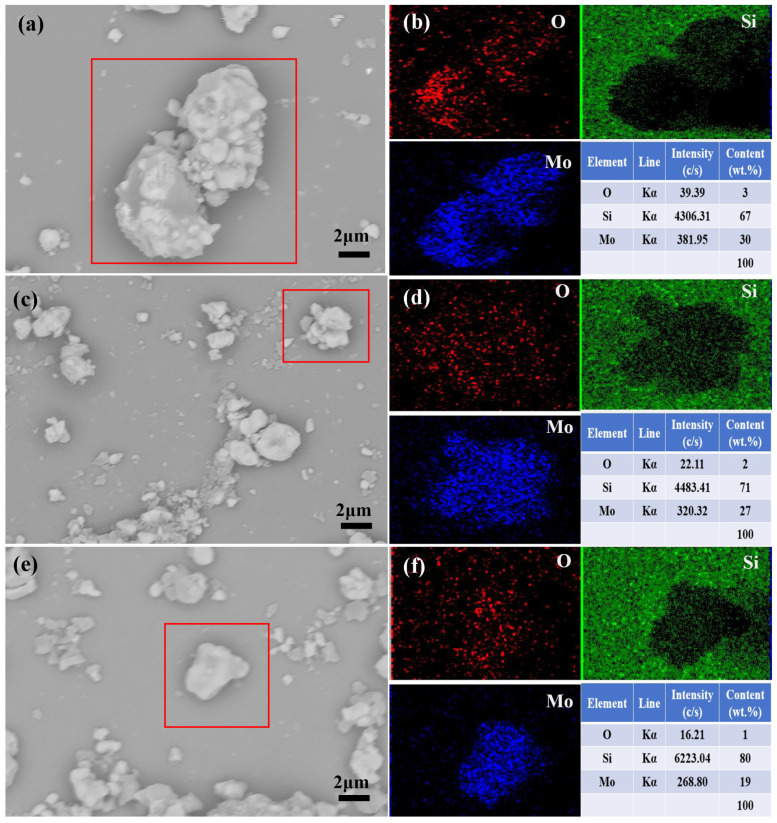
SEM with BSE imaging and EDS mapping of the particles in red squares with element contents of AS-MoSi_2_ (**a**,**b**), NS-MoSi_2_ (**c**,**d**), and BS-MoSi_2_ (**e**,**f**).

The surface elemental distributions of AS-MoSi_2_, NS-MoSi_2_, and BS-MoSi_2_ were investigated using EDS mapping in correlation with SEM morphology (Figure 5). The combined SEM with BSE imaging (Figure 5a,c,e) and EDS mapping (Figure 5b,d,f) data reveal distinct elemental distributions that correlate with microstructural evolution.

In AS-MoSi_2_ (Figure 5a,b), the SEM image reveals large particulate aggregates with rough surface texture, while EDS mapping shows intense red regions of oxygen, indicating localized oxygen enrichment. This corresponds to thick but uneven gel layer formation, likely due to heterogeneous nucleation during coating deposition. For NS-MoSi_2_ (Figure 5c,d), SEM imaging demonstrates finer particulate dispersion with reduced aggregation. The corresponding EDS mapping exhibits sparse red oxygen signals of oxygen, consistent with the lower surface charge density of MoSi_2_ in neutral conditions, which restricts effective SiO_2_ gel polymerization and leads to discontinuous coating formation. The BS-MoSi_2_ sample (Figure 5e,f) displays uniform surface morphology with a tightly packed surface in SEM imaging. EDS mapping confirms homogeneous elemental distribution, particularly notable for the significantly reduced molybdenum content (19 wt% vs. 30 wt% in AS-MoSi_2_). The notably high silicon content (80 wt%) suggests the effective suppression of oxidation through alkaline-induced Si–O–Si network densification, forming a conformal gel coating that minimizes MoSi_2_ exposure.

This systematic comparison demonstrates how pH-mediated sol-adsorption modulates both coating morphology and chemical composition, with alkaline conditions proving most effective for achieving homogeneous protective layers on MoSi_2_ surfaces.

**Figure 6:**
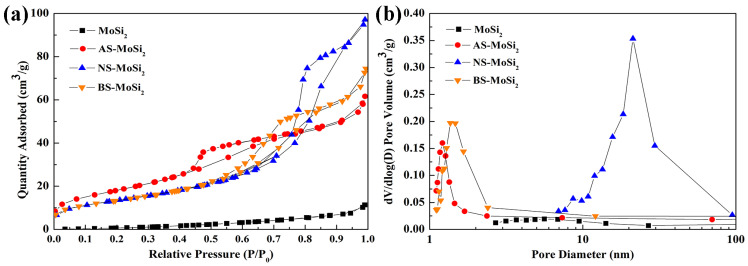
Nitrogen adsorption-desorption isotherms (**a**) and pore size distributions (**b**) of MoSi_2_, AS-MoSi_2_, NS-MoSi_2_, and BS-MoSi_2_.

To further investigate the pore structure of the samples, adsorption/desorption behaviors of MoSi_2_, AS-MoSi_2_, NS-MoSi_2_, and BS-MoSi_2_ were evaluated through the BET test. Figure 6 illustrates the nitrogen adsorption–desorption isotherms and pore size distributions of the samples. According to IUPAC classification, the curve of MoSi_2_ shows type III isotherm, and the curves of the AS-MoSi_2_, NS-MoSi_2_, and BS-MoSi_2_ show type IV isotherms with H2a hysteresis loops (Figure 6a). The IV curve indicates that the porous material has multiple adsorption forces on the surface, a pronounced pore structure, and an increase in adsorption rate at low relative pressures, indicating the presence of mesoporous structures within the samples (Figure 6b) [31].

Table 2 summarizes the detailed test data. BS-MoSi_2_ possesses the smallest pore size (2.86 nm) and the largest specific surface area (66.61 m^2^/g) among the three samples, resulting in poor oxidation resistance. NS-MoSi_2_ has similar surface areas but relatively larger pore sizes (6.11 nm) compared with BS-MoSi_2_ (4.72 nm), providing channels for oxygen to diffuse inward. Overall, BS-MoSi_2_ has the smallest specific surface area and middle pore size; therefore, it has the best oxidation resistance.

### 3.3. Mechanism Analysis of Coating Encapsulation of MoSi_2_

The coating formation mechanism can be elucidated through thermodynamic, kinetic, and surface interaction analyses. Thermodynamically, heterogeneous nucleation of silica sol particles on MoSi_2_ surfaces requires less surface energy than homogeneous nucleation in aqueous phases [32], establishing preferential surface coating. Three key factors govern this coating process: the dispersion and surface properties of MoSi_2_ itself, the colloidal stability of silica sol, and the reactivity of MoSi_2_ with silica sol.

Firstly, the isoelectric point of MoSi_2_ is between pH 1–2, and the zeta potential of MoSi_2_ becomes more negative with increasing pH, indicating that MoSi_2_ is negatively charged in all three types of silica sol. MoSi_2_ has poor dispersibility and is prone to aggregation in acidic environments, while in alkaline environments, the dispersibility is better and the particles are easier to be encapsulated [27].

Secondly, in terms of kinetics, the polymerization of silica sol involves competition between film-forming coating and nucleation coating [33]. That is, if the gel speed of silica sol is controlled, a uniform SiO_2_ film can be formed on the surface of MoSi_2_. If the polymerization of silica sol is too fast, it will lead to self-nucleation and the formation of nucleation coating. pH is a key factor affecting the gelation time of silica sol. When the concentration is the same, the curve of gelation time (*t*) with pH changes in an “N” shape [34]. The pH corresponding to the lg*t* of acidic silica sol and alkaline silica sol is located at the high point of the N-shaped curve, and the gelation speed of silica sol is slow, which is conducive to the formation of continuous and uniform film coating. The pH corresponding to the lg*t* of neutral silica sol is located at the low point of the N-shaped curve, with a fast gelation rate, which is not conducive to film coating and tends to self-nucleate.

Lastly, as mentioned earlier, in acidic environments, negatively charged MoSi_2_ and positively charged silica sol undergo electrostatic attraction, leading to rapid adsorption and deposition of silica sol on the surface of MoSi_2_ particles, resulting in high coating efficiency; in neutral environment, less surface groups lead to less condensation; and in basic environment, there is electrostatic repulsion between charges of the same type, but polymerization reactions can still occur.

**Figure 7:**
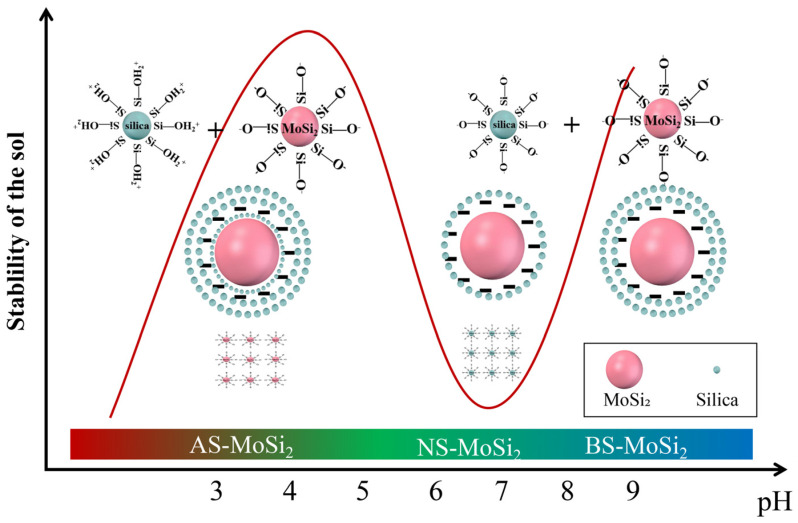
Schematic diagram of film formation mechanism of AS-MoSi_2_, NS-MoSi_2_ and BS-MoSi_2_.

Based on the above analysis, schematic diagrams of the film formation mechanism of AS-MoSi_2_, NS-MoSi_2_, and BS-MoSi_2_ are shown in Figure 7. Under acidic conditions (pH < 3), MoSi_2_ exhibits poor dispersibility due to electrostatic aggregation. Although rapid adsorption of silica sol particles (Si–OH_2_^+^) occurs via electrostatic attraction to the negatively charged MoSi_2_ surface (Si–O^−^), the high gelation rate promotes premature nucleation rather than uniform film growth. This results in thick but porous gel films with irregular coverage and limited oxidation resistance. In neutral silica sol, MoSi_2_ has enhanced dispersibility but fewer surface functional groups, which is not conducive to the formation of adsorption layers. At the same time, the silica sol has a fast gelation rate, resulting in the formation of SiO_2_ aggregates between particles. In basic silica sol, the good dispersibility of MoSi_2_, the electrostatic repulsion between particles, and the slow gelling speed of silica sol are conducive to the polymerization to form a uniform gel film.

The coating quality and oxidation resistance of BS-MoSi_2_ arise from optimal gelation kinetics under alkaline conditions (slow, homogeneous growth), enhanced surface coverage via reduced agglomeration in high-pH media, and suppressed premature nucleation through controlled charge interactions. This pH-dependent behavior provides a framework for optimizing sol-gel derived coatings, where colloidal stability and reaction kinetics are precisely balanced to achieve conformal encapsulation.

## 4. Conclusions

In this study, acidic, neutral, and basic silica sols were systematically applied to coat MoSi_2_ powders through sol-adsorption and encapsulation. Two distinct fabrication routes were employed: (1) ethanol-mediated dispersion of MoSi_2_ followed by silica sol impregnation, and (2) direct mixing of MoSi_2_ with silica sol. The impact of preparation methodology and silica sol pH on protective film characteristics and resulting oxidation resistance of core-shell structured MoSi_2_@SiO_2_ powders was systematically investigated. The main results are as follows.

(1) AS-MoSi_2_, NS-MoSi_2_, and BS-MoSi_2_ specimens exhibited significantly reduced coating efficiency values (maximum 27%) when fabricated via ethanol-mediated dispersion compared with direct mixing. All xerogel powder exhibited reduced mass gain compared to pristine MoSi_2_ in TG analysis, with ethanol-free preparations showing 10–20% lower weight gains between 340 °C and 600 °C than their ethanol-mediated dispersed counterparts. The NS-MoSi_2_ demonstrated delayed oxidation kinetics: oxidation initiation shifted from 340 °C (MoSi_2_) to 390 °C, with peak reaction rates occurring at 480 °C (vs. 450 °C for MoSi_2_).
(2) There is a clear correlation between the coating weight gain rate and the type of silica sol used. The AS-MoSi_2_ sample exhibits the highest coating weight gain rate at 9.12 ± 0.26%, followed by NS-MoSi_2_ with 8.60 ± 0.41%, while BS-MoSi_2_ demonstrates the lowest value at 7.74 ± 0.06%. While the weight gains between 340 °C and 600 °C due to oxidation were quantified as 31.58%, 28.34%, and 18.45% for AS-MoSi_2_, NS-MoSi_2_, and BS-MoSi_2_, respectively, indicate that BS-MoSi_2_ exhibits superior oxidant resistance.
(3) The oxidation resistance of BS-MoSi_2_ arises from the coating quality, which is determined by optimal gelation kinetics under alkaline conditions (slow, homogeneous growth); enhanced surface coverage via reduced agglomeration in high-pH media; and suppressed premature nucleation through controlled charge interactions.

## Figures and Tables

**Table 1:** Parameters of the silica sol.

Type	pH	SiO_2_ Content (%)	Na_2_O Content (%)	Density (20 °C, g·cm^−3^)	Viscosity (20 °C, mPas)
Acid silica sol	2.92 ± 0.01	30 ± 0.1	≤0.06	1.17 ± 0.01	≤7
Neutral silica sol	7.31 ± 0.01	32 ± 0.1	≤0.1	1.18 ± 0.01	≤7
Alkaline silica sol	9.49 ± 0.01	32 ± 0.1	≤0.3	1.20 ± 0.01	≤10

**Table 2:** Parameters of MoSi_2_, AS-MoSi_2_, NS-MoSi_2_, and BS-MoSi_2_.

Type	MoSi_2_	AS-MoSi_2_	NS-MoSi_2_	BS-MoSi_2_
BET surface area (m^2^/g)	6.15	66.61	49.17	48.75
Total pore volume of adsorption (cm^3^/g)	0.021	0.102	0.160	0.126
Pore size (nm)	5.72	2.86	6.11	4.72

## Data Availability

The original contributions presented in this study are included in the article. Further inquiries can be directed to the corresponding authors.
